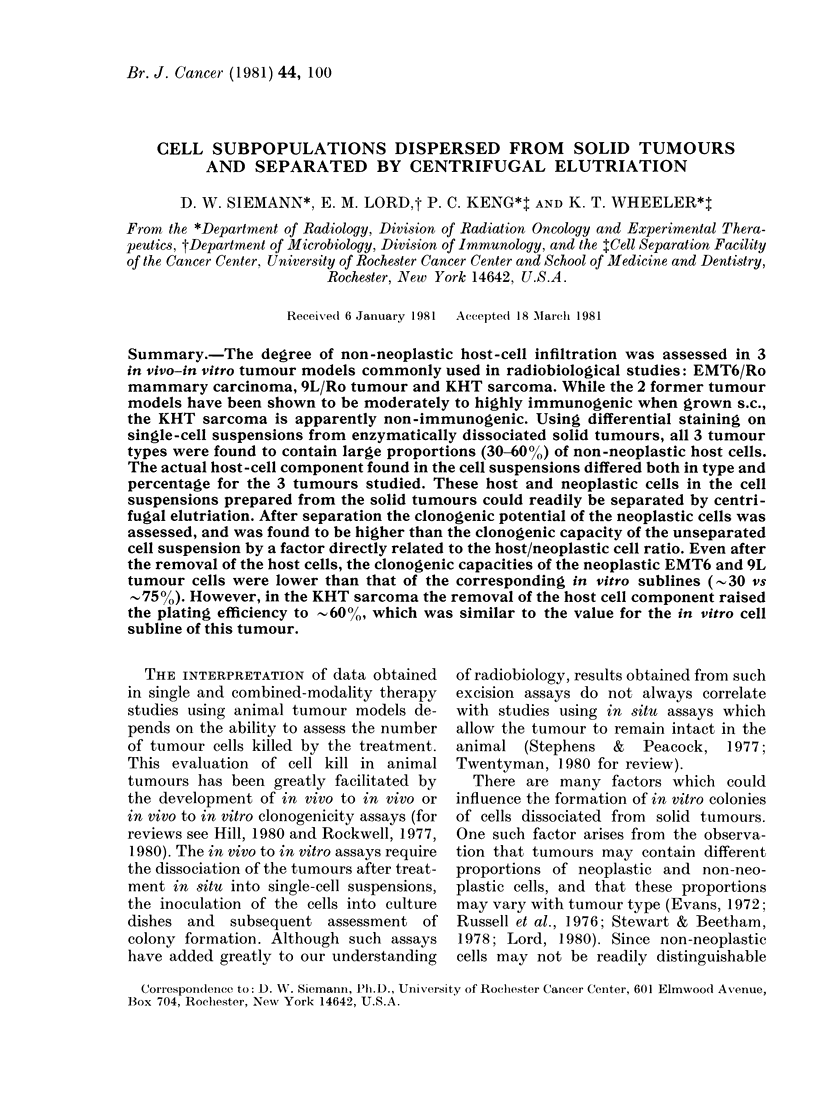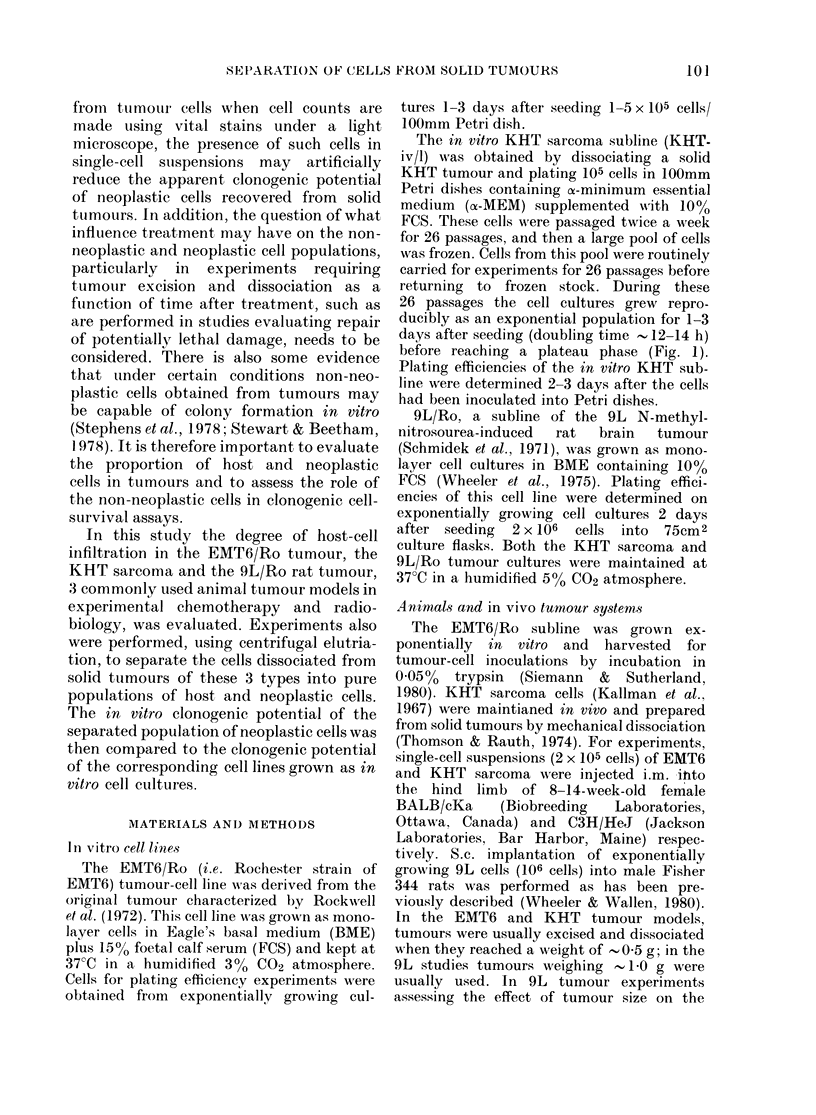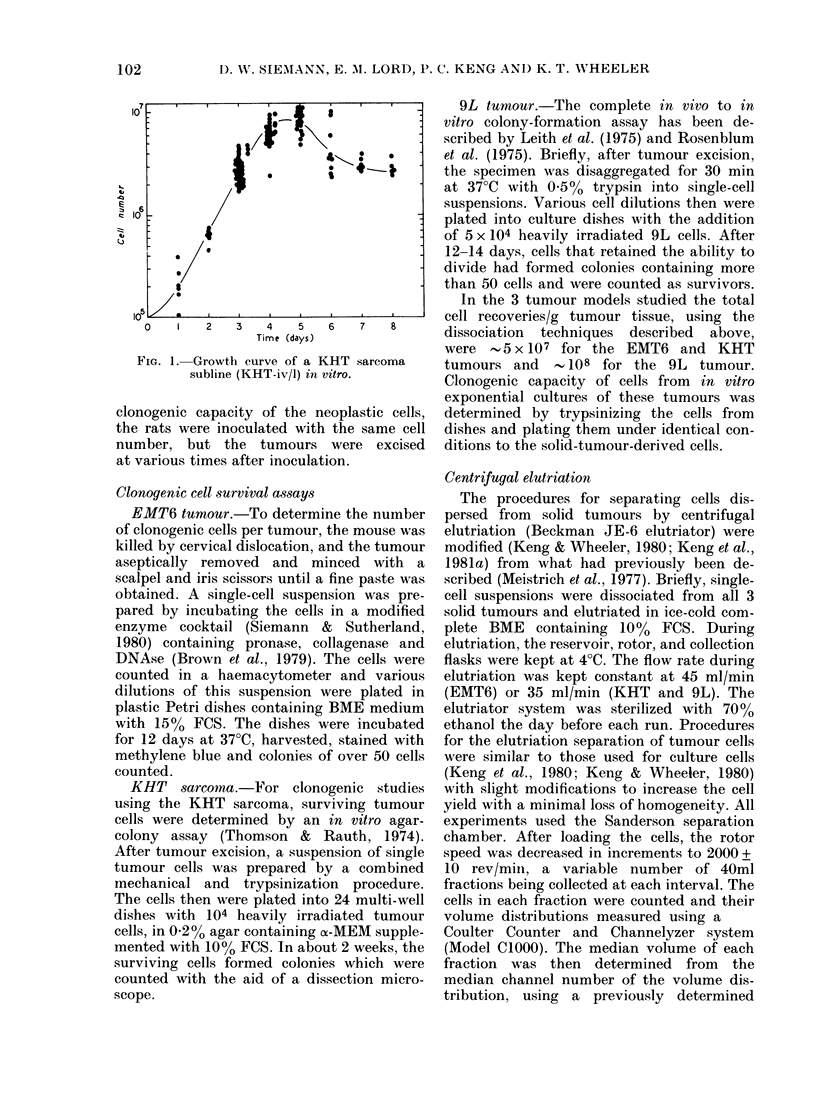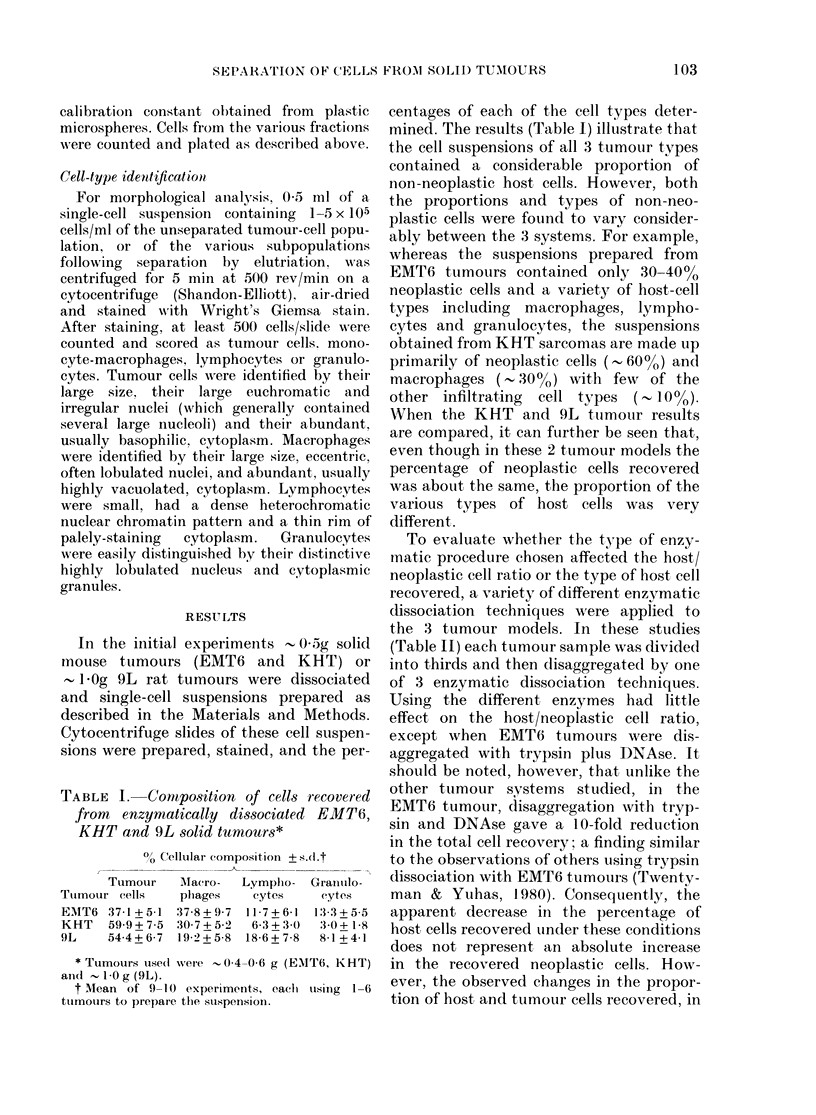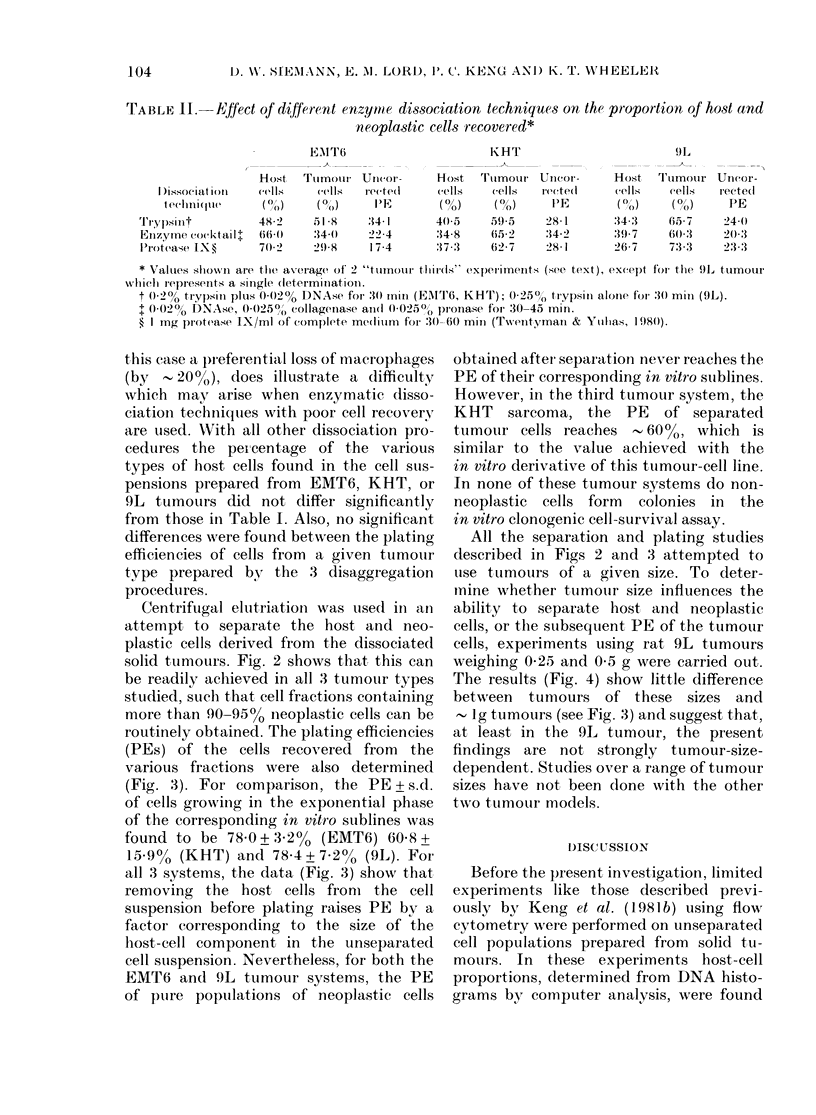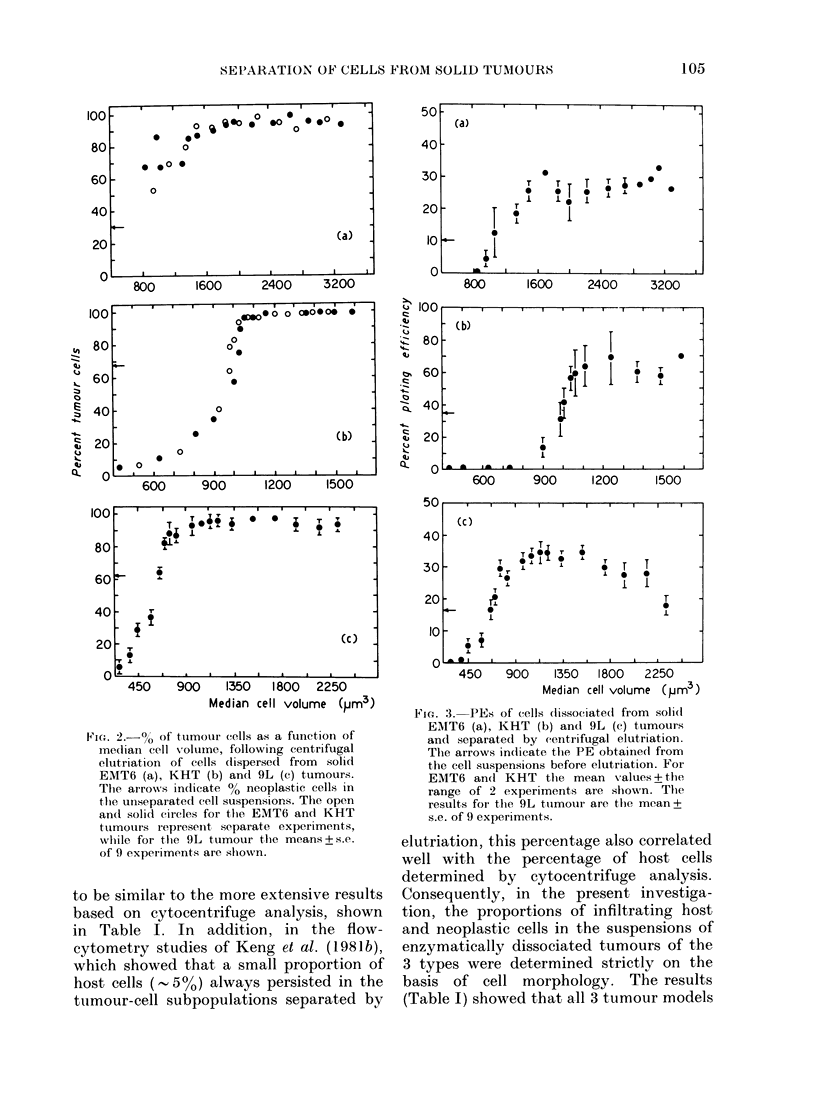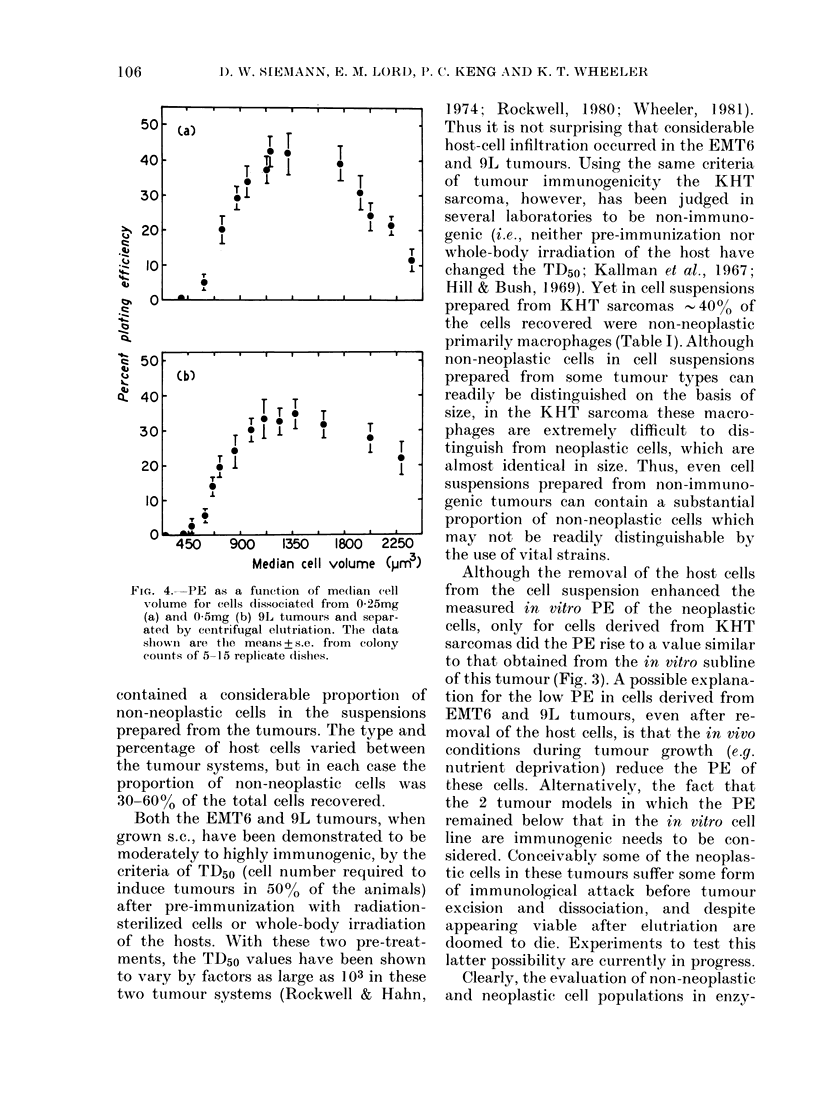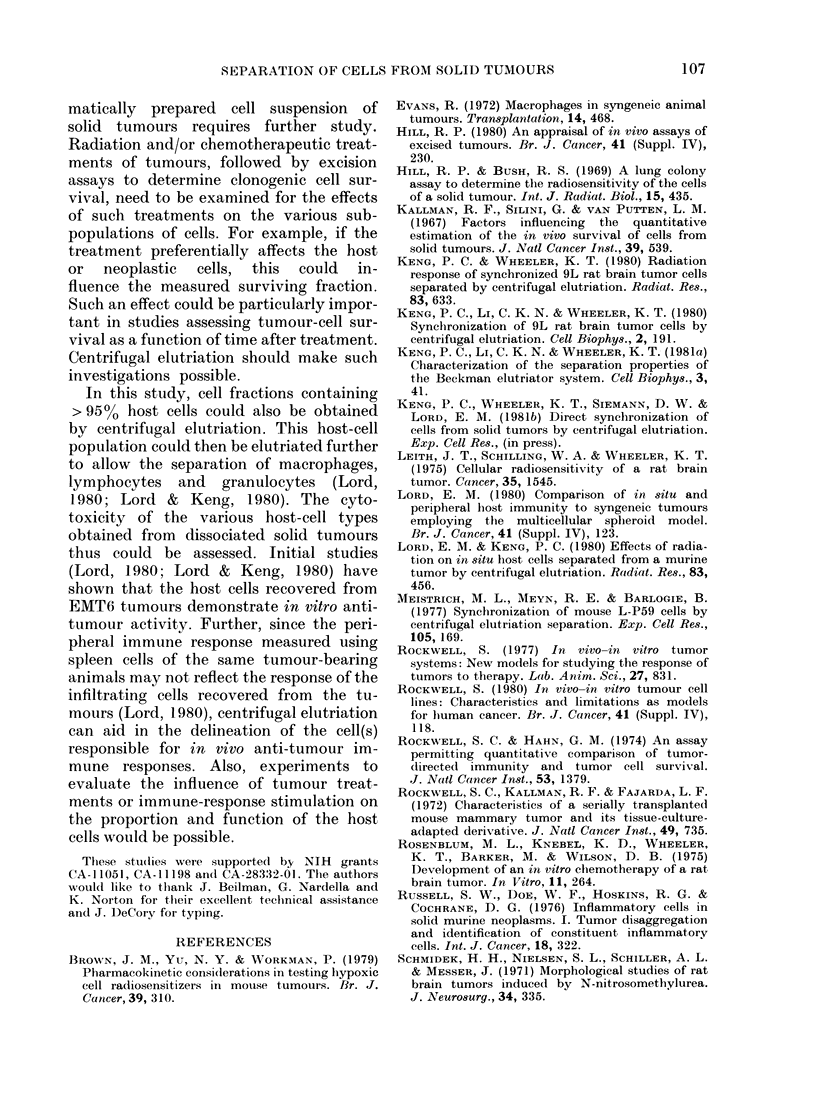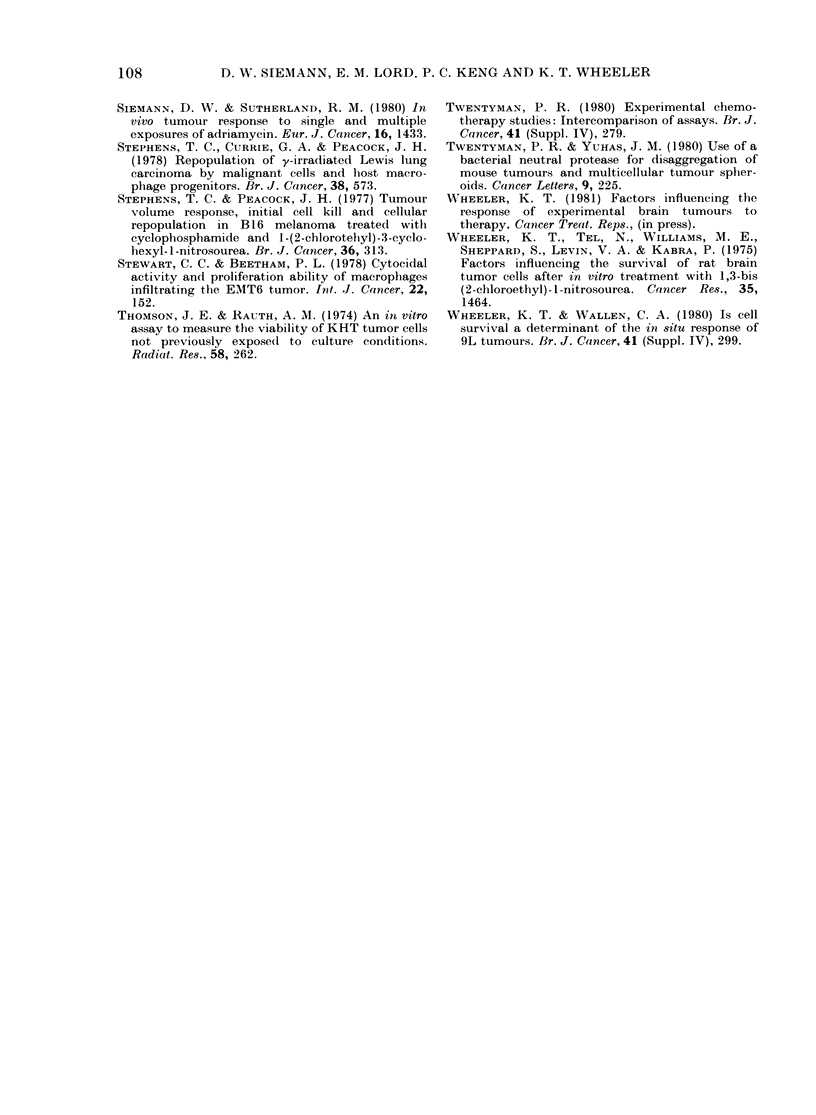# Cell subpopulations dispersed from solid tumours and separated by centrifugal elutriation.

**DOI:** 10.1038/bjc.1981.154

**Published:** 1981-07

**Authors:** D. W. Siemann, E. M. Lord, P. C. Keng, K. T. Wheeler

## Abstract

The degree of non-neoplastic host-cell infiltration was assessed in 3 in vivo-in vitro tumour models commonly used in radiobiological studies: EMT6/Ro mammary carcinoma, 9L/Ro tumour and KHT sarcoma. While the 2 former tumour models have been shown to be moderately to highly immunogenic when grown s.c., the KHT sarcoma is apparently non-immunogenic. Using differential staining on single-cell suspensions from enzymatically dissociated solid tumours, all 3 tumour types were found to contain large proportions (30-60%) of non-neoplastic host cells. The actual host-cell component found in the cell suspensions differed both in type and percentage for the 3 tumours studied. These host and neoplastic cells in the cell suspensions prepared from the solid tumours could be readily separated by centrifugal elutriation. After separation the clonogenic potential of the neoplastic cells was assessed, and was found to be higher than the clonogenic capacity of the unseparated cell suspension by a factor directly related to the host/neoplastic cell ratio. Even after the removal of the host cells, the clonogenic capacities of the neoplastic EMT6 and 9L tumour cells were lower than that of the corresponding in vitro sublines (approximately 30 vs 75%). However, in the KHT sarcoma the removal of the host cell component raised the plating efficiency to approximately 60%, which was similar to the value for the in vitro cell subline of this tumour.


*Br. J. Cancer* (1981) **44**, 100

### CELL SUBPOPULATIONS DISPERSED FROM SOLID TUMOURS AND SEPARATED BY CENTRIFUGAL ELUTRIATION

D. W. SIEMANN*, E. M. LORD,† P. C. KENG*‡ AND K. T. WHEELER*‡

*From the *Department of Radiology, Division of Radiation Oncology and Experimental Therapeutics, †Department of Microbiology, Division of Immunology, and the ‡Cell Separation Facility of the Cancer Center, University of Rochester Cancer Center and School of Medicine and Dentistry, Rochester, New York* 14642, *U.S.A.*

Received 6 January 1981 Accepted 18 March 1981

**Summary.—The degree of non-neoplastic host-cell infiltration was assessed in 3 *in vivo–in vitro* tumour models commonly used in radiobiological studies: EMT6/Ro mammary carcinoma, 9L/Ro tumour and KHT sarcoma. While the 2 former tumour models have been shown to be moderately to highly immunogenic when grown s.c., the KHT sarcoma is apparently non-immunogenic. Using differential staining on single-cell suspensions from enzymatically dissociated solid tumours, all 3 tumour types were found to contain large proportions (30–60%) of non-neoplastic host cells. The actual host-cell component found in the cell suspensions differed both in type and percentage for the 3 tumours studied. These host and neoplastic cells in the cell suspensions prepared from the solid tumours could readily be separated by centrifugal elutriation. After separation the clonogenic potential of the neoplastic cells was assessed, and was found to be higher than the clonogenic capacity of the unseparated cell suspension by a factor directly related to the host/neoplastic cell ratio. Even after the removal of the host cells, the clonogenic capacities of the neoplastic EMT6 and 9L tumour cells were lower than that of the corresponding *in vitro* sublines (~30 *vs* ~75%). However, in the KHT sarcoma the removal of the host cell component raised the plating efficiency to ~60%, which was similar to the value for the *in vitro* cell subline of this tumour.**

THE INTERPRETATION of data obtained in single and combined-modality therapy studies using animal tumour models depends on the ability to assess the number of tumour cells killed by the treatment. This evaluation of cell kill in animal tumours has been greatly facilitated by the development of *in vivo* to *in vivo* or *in vivo* to *in vitro* clonogenicity assays (for reviews see Hill, 1980 and Rockwell, 1977, 1980). The *in vivo* to *in vitro* assays require the dissociation of the tumours after treatment *in situ* into single-cell suspensions, the inoculation of the cells into culture dishes and subsequent assessment of colony formation. Although such assays have added greatly to our understanding of radiobiology, results obtained from such excision assays do not always correlate with studies using *in situ* assays which allow the tumour to remain intact in the animal (Stephens & Peacock, 1977; Twentyman, 1980 for review).

There are many factors which could influence the formation of *in vitro* colonies of cells dissociated from solid tumours. One such factor arises from the observation that tumours may contain different proportions of neoplastic and non-neoplastic cells, and that these proportions may vary with tumour type (Evans, 1972; Russell *et al.*, 1976; Stewart & Beetham, 1978; Lord, 1980). Since non-neoplastic cells may not be readily distinguishable

Correspondence to: D. W. Siemann, Ph.D., University of Rochester Cancer Center, 601 Elmwood Avenue, Box 704, Rochester, New York 14642, U.S.A.

from tumour cells when cell counts are made using vital stains under a light microscope, the presence of such cells in single-cell suspensions may artificially reduce the apparent clonogenic potential of neoplastic cells recovered from solid tumours. In addition, the question of what influence treatment may have on the non-neoplastic and neoplastic cell populations, particularly in experiments requiring tumour excision and dissociation as a function of time after treatment, such as are performed in studies evaluating repair of potentially lethal damage, needs to be considered. There is also some evidence that under certain conditions non-neoplastic cells obtained from tumours may be capable of colony formation *in vitro* (Stephens *et al.*, 1978; Stewart & Beetham, 1978). It is therefore important to evaluate the proportion of host and neoplastic cells in tumours and to assess the role of the non-neoplastic cells in clonogenic cell-survival assays.

In this study the degree of host-cell infiltration in the EMT6/Ro tumour, the KHT sarcoma and the 9L/Ro rat tumour, 3 commonly used animal tumour models in experimental chemotherapy and radiobiology, was evaluated. Experiments also were performed, using centrifugal elutriation, to separate the cells dissociated from solid tumours of these 3 types into pure populations of host and neoplastic cells. The *in vitro* clonogenic potential of the separated population of neoplastic cells was then compared to the clonogenic potential of the corresponding cell lines grown as *in vitro* cell cultures.

### MATERIALS AND METHODS

### In vitro *cell lines*

The EMT6/Ro (*i.e.* Rochester strain of EMT6) tumour-cell line was derived from the original tumour characterized by Rockwell *et al.* (1972). This cell line was grown as monolayer cells in Eagle's basal medium (BME) plus 15% foetal calf serum (FCS) and kept at 37°C in a humidified 3% $CO_2$ atmosphere. Cells for plating efficiency experiments were obtained from exponentially growing cultures 1–3 days after seeding $1–5 \times 10^5$ cells/100mm Petri dish.

The *in vitro* KHT sarcoma subline (KHT-iv/1) was obtained by dissociating a solid KHT tumour and plating $10^5$ cells in 100mm Petri dishes containing α-minimum essential medium (α-MEM) supplemented with 10% FCS. These cells were passaged twice a week for 26 passages, and then a large pool of cells was frozen. Cells from this pool were routinely carried for experiments for 26 passages before returning to frozen stock. During these 26 passages the cell cultures grew reproducibly as an exponential population for 1–3 days after seeding (doubling time ~12–14 h) before reaching a plateau phase (Fig. 1). Plating efficiencies of the *in vitro* KHT subline were determined 2–3 days after the cells had been inoculated into Petri dishes.

9L/Ro, a subline of the 9L N-methyl-nitrosourea-induced rat brain tumour (Schmidek *et al.*, 1971), was grown as monolayer cell cultures in BME containing 10% FCS (Wheeler *et al.*, 1975). Plating efficiencies of this cell line were determined on exponentially growing cell cultures 2 days after seeding $2 \times 10^6$ cells into 75cm$^2$ culture flasks. Both the KHT sarcoma and 9L/Ro tumour cultures were maintained at 37°C in a humidified 5% $CO_2$ atmosphere.

### *Animals and* in vivo *tumour systems*

The EMT6/Ro subline was grown exponentially *in vitro* and harvested for tumour-cell inoculations by incubation in 0·05% trypsin (Siemann & Sutherland, 1980). KHT sarcoma cells (Kallman *et al.*, 1967) were maintianed *in vivo* and prepared from solid tumours by mechanical dissociation (Thomson & Rauth, 1974). For experiments, single-cell suspensions ($2 \times 10^5$ cells) of EMT6 and KHT sarcoma were injected i.m. into the hind limb of 8–14-week-old female BALB/cKa (Biobreeding Laboratories, Ottawa, Canada) and C3H/HeJ (Jackson Laboratories, Bar Harbor, Maine) respectively. S.c. implantation of exponentially growing 9L cells ($10^6$ cells) into male Fisher 344 rats was performed as has been previously described (Wheeler & Wallen, 1980). In the EMT6 and KHT tumour models, tumours were usually excised and dissociated when they reached a weight of ~0·5 g; in the 9L studies tumours weighing ~1·0 g were usually used. In 9L tumour experiments assessing the effect of tumour size on the

FIG. 1.—Growth curve of a KHT sarcoma subline (KHT-iv/l) *in vitro*.

clonogenic capacity of the neoplastic cells, the rats were inoculated with the same cell number, but the tumours were excised at various times after inoculation.

*Clonogenic cell survival assays*

*EMT6 tumour.*—To determine the number of clonogenic cells per tumour, the mouse was killed by cervical dislocation, and the tumour aseptically removed and minced with a scalpel and iris scissors until a fine paste was obtained. A single-cell suspension was prepared by incubating the cells in a modified enzyme cocktail (Siemann & Sutherland, 1980) containing pronase, collagenase and DNAse (Brown *et al.*, 1979). The cells were counted in a haemacytometer and various dilutions of this suspension were plated in plastic Petri dishes containing BME medium with 15% FCS. The dishes were incubated for 12 days at 37°C, harvested, stained with methylene blue and colonies of over 50 cells counted.

*KHT sarcoma.*—For clonogenic studies using the KHT sarcoma, surviving tumour cells were determined by an *in vitro* agar-colony assay (Thomson & Rauth, 1974). After tumour excision, a suspension of single tumour cells was prepared by a combined mechanical and trypsinization procedure. The cells then were plated into 24 multi-well dishes with $10^4$ heavily irradiated tumour cells, in 0·2% agar containing α-MEM supplemented with 10% FCS. In about 2 weeks, the surviving cells formed colonies which were counted with the aid of a dissection microscope.

*9L tumour.*—The complete *in vivo* to *in vitro* colony-formation assay has been described by Leith *et al.* (1975) and Rosenblum *et al.* (1975). Briefly, after tumour excision, the specimen was disaggregated for 30 min at 37°C with 0·5% trypsin into single-cell suspensions. Various cell dilutions then were plated into culture dishes with the addition of $5 \times 10^4$ heavily irradiated 9L cells. After 12–14 days, cells that retained the ability to divide had formed colonies containing more than 50 cells and were counted as survivors.

In the 3 tumour models studied the total cell recoveries/g tumour tissue, using the dissociation techniques described above, were $\sim 5 \times 10^7$ for the EMT6 and KHT tumours and $\sim 10^8$ for the 9L tumour. Clonogenic capacity of cells from *in vitro* exponential cultures of these tumours was determined by trypsinizing the cells from dishes and plating them under identical conditions to the solid-tumour-derived cells.

*Centrifugal elutriation*

The procedures for separating cells dispersed from solid tumours by centrifugal elutriation (Beckman JE-6 elutriator) were modified (Keng & Wheeler, 1980; Keng *et al.*, 1981*a*) from what had previously been described (Meistrich *et al.*, 1977). Briefly, single-cell suspensions were dissociated from all 3 solid tumours and elutriated in ice-cold complete BME containing 10% FCS. During elutriation, the reservoir, rotor, and collection flasks were kept at 4°C. The flow rate during elutriation was kept constant at 45 ml/min (EMT6) or 35 ml/min (KHT and 9L). The elutriator system was sterilized with 70% ethanol the day before each run. Procedures for the elutriation separation of tumour cells were similar to those used for culture cells (Keng *et al.*, 1980; Keng & Wheeler, 1980) with slight modifications to increase the cell yield with a minimal loss of homogeneity. All experiments used the Sanderson separation chamber. After loading the cells, the rotor speed was decreased in increments to 2000 ± 10 rev/min, a variable number of 40ml fractions being collected at each interval. The cells in each fraction were counted and their volume distributions measured using a Coulter Counter and Channelyzer system (Model C1000). The median volume of each fraction was then determined from the median channel number of the volume distribution, using a previously determined calibration constant obtained from plastic microspheres. Cells from the various fractions were counted and plated as described above.

*Cell-type identification*

For morphological analysis, 0·5 ml of a single-cell suspension containing $1–5 \times 10^5$ cells/ml of the unseparated tumour-cell population, or of the various subpopulations following separation by elutriation, was centrifuged for 5 min at 500 rev/min on a cytocentrifuge (Shandon-Elliott), air-dried and stained with Wright's Giemsa stain. After staining, at least 500 cells/slide were counted and scored as tumour cells, monocyte-macrophages, lymphocytes or granulocytes. Tumour cells were identified by their large size, their large euchromatic and irregular nuclei (which generally contained several large nucleoli) and their abundant, usually basophilic, cytoplasm. Macrophages were identified by their large size, eccentric, often lobulated nuclei, and abundant, usually highly vacuolated, cytoplasm. Lymphocytes were small, had a dense heterochromatic nuclear chromatin pattern and a thin rim of palely-staining cytoplasm. Granulocytes were easily distinguished by their distinctive highly lobulated nucleus and cytoplasmic granules.

### RESULTS

In the initial experiments ~0·5g solid mouse tumours (EMT6 and KHT) or ~1·0g 9L rat tumours were dissociated and single-cell suspensions prepared as described in the Materials and Methods. Cytocentrifuge slides of these cell suspensions were prepared, stained, and the percentages of each of the cell types determined. The results (Table I) illustrate that the cell suspensions of all 3 tumour types contained a considerable proportion of non-neoplastic host cells. However, both the proportions and types of non-neoplastic cells were found to vary considerably between the 3 systems. For example, whereas the suspensions prepared from EMT6 tumours contained only 30–40% neoplastic cells and a variety of host-cell types including macrophages, lymphocytes and granulocytes, the suspensions obtained from KHT sarcomas are made up primarily of neoplastic cells (~60%) and macrophages (~30%) with few of the other infiltrating cell types (~10%). When the KHT and 9L tumour results are compared, it can further be seen that, even though in these 2 tumour models the percentage of neoplastic cells recovered was about the same, the proportion of the various types of host cells was very different.

To evaluate whether the type of enzymatic procedure chosen affected the host/neoplastic cell ratio or the type of host cell recovered, a variety of different enzymatic dissociation techniques were applied to the 3 tumour models. In these studies (Table II) each tumour sample was divided into thirds and then disaggregated by one of 3 enzymatic dissociation techniques. Using the different enzymes had little effect on the host/neoplastic cell ratio, except when EMT6 tumours were disaggregated with trypsin plus DNAse. It should be noted, however, that unlike the other tumour systems studied, in the EMT6 tumour, disaggregation with trypsin and DNAse gave a 10-fold reduction in the total cell recovery; a finding similar to the observations of others using trypsin dissociation with EMT6 tumours (Twentyman & Yuhas, 1980). Consequently, the apparent decrease in the percentage of host cells recovered under these conditions does not represent an absolute increase in the recovered neoplastic cells. However, the observed changes in the proportion of host and tumour cells recovered, in

TABLE I.—*Composition of cells recovered from enzymatically dissociated EMT6, KHT and 9L solid tumours**

| | % Cellular composition ± s.d.† | | | |
|---|---|---|---|---|
| Tumour | Tumour cells | Macrophages | Lymphocytes | Granulocytes |
| EMT6 | 37·1 ± 5·1 | 37·8 ± 9·7 | 11·7 ± 6·1 | 13·3 ± 5·5 |
| KHT | 59·9 ± 7·5 | 30·7 ± 5·2 | 6·3 ± 3·0 | 3·0 ± 1·8 |
| 9L | 54·4 ± 6·7 | 19·2 ± 5·8 | 18·6 ± 7·8 | 8·1 ± 4·1 |

* Tumours used were ~0·4–0·6 g (EMT6, KHT) and ~1·0 g (9L).

† Mean of 9–10 experiments, each using 1–6 tumours to prepare the suspension.

TABLE II.—*Effect of different enzyme dissociation techniques on the proportion of host and neoplastic cells recovered**

| | EMT6 | | | KHT | | | 9L | | |
|---|---|---|---|---|---|---|---|---|---|
| Dissociation technique | Host cells (%) | Tumour cells (%) | Uncorrected PE | Host cells (%) | Tumour cells (%) | Uncorrected PE | Host cells (%) | Tumour cells (%) | Uncorrected PE |
| Trypsin† | 48·2 | 51·8 | 34·1 | 40·5 | 59·5 | 28·1 | 34·3 | 65·7 | 24·0 |
| Enzyme cocktail‡ | 66·0 | 34·0 | 22·4 | 34·8 | 65·2 | 34·2 | 39·7 | 60·3 | 20·3 |
| Protease IX§ | 70·2 | 29·8 | 17·4 | 37·3 | 62·7 | 28·1 | 26·7 | 73·3 | 23·3 |

* Values shown are the average of 2 "tumour thirds" experiments (see text), except for the 9L tumour which represents a single determination.

† 0·2% trypsin plus 0·02% DNAse for 30 min (EMT6, KHT); 0·25% trypsin alone for 30 min (9L).

‡ 0·02% DNAse, 0·025% collagenase and 0·025% pronase for 30–45 min.

§ 1 mg protease IX/ml of complete medium for 30–60 min (Twentyman & Yuhas, 1980).

this case a preferential loss of macrophages (by ~20%), does illustrate a difficulty which may arise when enzymatic dissociation techniques with poor cell recovery are used. With all other dissociation procedures the percentage of the various types of host cells found in the cell suspensions prepared from EMT6, KHT, or 9L tumours did not differ significantly from those in Table I. Also, no significant differences were found between the plating efficiencies of cells from a given tumour type prepared by the 3 disaggregation procedures.

Centrifugal elutriation was used in an attempt to separate the host and neoplastic cells derived from the dissociated solid tumours. Fig. 2 shows that this can be readily achieved in all 3 tumour types studied, such that cell fractions containing more than 90–95% neoplastic cells can be routinely obtained. The plating efficiencies (PEs) of the cells recovered from the various fractions were also determined (Fig. 3). For comparison, the PE ± s.d. of cells growing in the exponential phase of the corresponding *in vitro* sublines was found to be 78·0 ± 3·2% (EMT6) 60·8 ± 15·9% (KHT) and 78·4 ± 7·2% (9L). For all 3 systems, the data (Fig. 3) show that removing the host cells from the cell suspension before plating raises PE by a factor corresponding to the size of the host-cell component in the unseparated cell suspension. Nevertheless, for both the EMT6 and 9L tumour systems, the PE of pure populations of neoplastic cells obtained after separation never reaches the PE of their corresponding *in vitro* sublines. However, in the third tumour system, the KHT sarcoma, the PE of separated tumour cells reaches ~60%, which is similar to the value achieved with the *in vitro* derivative of this tumour-cell line. In none of these tumour systems do non-neoplastic cells form colonies in the *in vitro* clonogenic cell-survival assay.

All the separation and plating studies described in Figs 2 and 3 attempted to use tumours of a given size. To determine whether tumour size influences the ability to separate host and neoplastic cells, or the subsequent PE of the tumour cells, experiments using rat 9L tumours weighing 0·25 and 0·5 g were carried out. The results (Fig. 4) show little difference between tumours of these sizes and ~1g tumours (see Fig. 3) and suggest that, at least in the 9L tumour, the present findings are not strongly tumour-size-dependent. Studies over a range of tumour sizes have not been done with the other two tumour models.

### DISCUSSION

Before the present investigation, limited experiments like those described previously by Keng *et al.* (1981*b*) using flow cytometry were performed on unseparated cell populations prepared from solid tumours. In these experiments host-cell proportions, determined from DNA histograms by computer analysis, were found

FIG. 2.—% of tumour cells as a function of median cell volume, following centrifugal elutriation of cells dispersed from solid EMT6 (a), KHT (b) and 9L (c) tumours. The arrows indicate % neoplastic cells in the unseparated cell suspensions. The open and solid circles for the EMT6 and KHT tumours represent separate experiments, while for the 9L tumour the means ± s.e. of 9 experiments are shown.

FIG. 3.—PEs of cells dissociated from solid EMT6 (a), KHT (b) and 9L (c) tumours and separated by centrifugal elutriation. The arrows indicate the PE obtained from the cell suspensions before elutriation. For EMT6 and KHT the mean values ± the range of 2 experiments are shown. The results for the 9L tumour are the mean ± s.e. of 9 experiments.

to be similar to the more extensive results based on cytocentrifuge analysis, shown in Table I. In addition, in the flow-cytometry studies of Keng *et al.* (1981*b*), which showed that a small proportion of host cells (~5%) always persisted in the tumour-cell subpopulations separated by elutriation, this percentage also correlated well with the percentage of host cells determined by cytocentrifuge analysis. Consequently, in the present investigation, the proportions of infiltrating host and neoplastic cells in the suspensions of enzymatically dissociated tumours of the 3 types were determined strictly on the basis of cell morphology. The results (Table I) showed that all 3 tumour models

FIG. 4.—PE as a function of median cell volume for cells dissociated from 0·25mg (a) and 0·5mg (b) 9L tumours and separated by centrifugal elutriation. The data shown are the means ± s.e. from colony counts of 5–15 replicate dishes.

contained a considerable proportion of non-neoplastic cells in the suspensions prepared from the tumours. The type and percentage of host cells varied between the tumour systems, but in each case the proportion of non-neoplastic cells was 30–60% of the total cells recovered.

Both the EMT6 and 9L tumours, when grown s.c., have been demonstrated to be moderately to highly immunogenic, by the criteria of $TD_{50}$ (cell number required to induce tumours in 50% of the animals) after pre-immunization with radiation-sterilized cells or whole-body irradiation of the hosts. With these two pre-treatments, the $TD_{50}$ values have been shown to vary by factors as large as $10^3$ in these two tumour systems (Rockwell & Hahn, 1974; Rockwell, 1980; Wheeler, 1981). Thus it is not surprising that considerable host-cell infiltration occurred in the EMT6 and 9L tumours. Using the same criteria of tumour immunogenicity the KHT sarcoma, however, has been judged in several laboratories to be non-immunogenic (*i.e.*, neither pre-immunization nor whole-body irradiation of the host have changed the $TD_{50}$; Kallman *et al.*, 1967; Hill & Bush, 1969). Yet in cell suspensions prepared from KHT sarcomas ~40% of the cells recovered were non-neoplastic primarily macrophages (Table I). Although non-neoplastic cells in cell suspensions prepared from some tumour types can readily be distinguished on the basis of size, in the KHT sarcoma these macrophages are extremely difficult to distinguish from neoplastic cells, which are almost identical in size. Thus, even cell suspensions prepared from non-immunogenic tumours can contain a substantial proportion of non-neoplastic cells which may not be readily distinguishable by the use of vital strains.

Although the removal of the host cells from the cell suspension enhanced the measured *in vitro* PE of the neoplastic cells, only for cells derived from KHT sarcomas did the PE rise to a value similar to that obtained from the *in vitro* subline of this tumour (Fig. 3). A possible explanation for the low PE in cells derived from EMT6 and 9L tumours, even after removal of the host cells, is that the *in vivo* conditions during tumour growth (*e.g.* nutrient deprivation) reduce the PE of these cells. Alternatively, the fact that the 2 tumour models in which the PE remained below that in the *in vitro* cell line are immunogenic needs to be considered. Conceivably some of the neoplastic cells in these tumours suffer some form of immunological attack before tumour excision and dissociation, and despite appearing viable after elutriation are doomed to die. Experiments to test this latter possibility are currently in progress.

Clearly, the evaluation of non-neoplastic and neoplastic cell populations in enzymatically prepared cell suspension of solid tumours requires further study. Radiation and/or chemotherapeutic treatments of tumours, followed by excision assays to determine clonogenic cell survival, need to be examined for the effects of such treatments on the various subpopulations of cells. For example, if the treatment preferentially affects the host or neoplastic cells, this could influence the measured surviving fraction. Such an effect could be particularly important in studies assessing tumour-cell survival as a function of time after treatment. Centrifugal elutriation should make such investigations possible.

In this study, cell fractions containing >95% host cells could also be obtained by centrifugal elutriation. This host-cell population could then be elutriated further to allow the separation of macrophages, lymphocytes and granulocytes (Lord, 1980; Lord & Keng, 1980). The cytotoxicity of the various host-cell types obtained from dissociated solid tumours thus could be assessed. Initial studies (Lord, 1980; Lord & Keng, 1980) have shown that the host cells recovered from EMT6 tumours demonstrate *in vitro* anti-tumour activity. Further, since the peripheral immune response measured using spleen cells of the same tumour-bearing animals may not reflect the response of the infiltrating cells recovered from the tumours (Lord, 1980), centrifugal elutriation can aid in the delineation of the cell(s) responsible for *in vivo* anti-tumour immune responses. Also, experiments to evaluate the influence of tumour treatments or immune-response stimulation on the proportion and function of the host cells would be possible.

These studies were supported by NIH grants CA-11051, CA-11198 and CA-28332-01. The authors would like to thank J. Beilman, G. Nardella and K. Norton for their excellent technical assistance and J. DeCory for typing.

### REFERENCES

BROWN, J. M., YU, N. Y. & WORKMAN, P. (1979) Pharmacokinetic considerations in testing hypoxic cell radiosensitizers in mouse tumours. *Br. J. Cancer*, **39**, 310.

EVANS, R. (1972) Macrophages in syngeneic animal tumours. *Transplantation*, **14**, 468.

HILL, R. P. (1980) An appraisal of *in vivo* assays of excised tumours. *Br. J. Cancer*, **41** (Suppl. IV), 230.

HILL, R. P. & BUSH, R. S. (1969) A lung colony assay to determine the radiosensitivity of the cells of a solid tumour. *Int. J. Radiat. Biol.*, **15**, 435.

KALLMAN, R. F., SILINI, G. & VAN PUTTEN, L. M. (1967) Factors influencing the quantitative estimation of the *in vivo* survival of cells from solid tumours. *J. Natl Cancer Inst.*, **39**, 539.

KENG, P. C. & WHEELER, K. T. (1980) Radiation response of synchronized 9L rat brain tumor cells separated by centrifugal elutriation. *Radiat. Res.*, **83**, 633.

KENG, P. C., LI, C. K. N. & WHEELER, K. T. (1980) Synchronization of 9L rat brain tumor cells by centrifugal elutriation. *Cell Biophys.*, **2**, 191.

KENG, P. C., LI, C. K. N. & WHEELER, K. T. (1981*a*) Characterization of the separation properties of the Beckman elutriator system. *Cell Biophys.*, **3**, 41.

KENG, P. C., WHEELER, K. T., SIEMANN, D. W. & LORD, E. M. (1981*b*) Direct synchronization of cells from solid tumors by centrifugal elutriation. *Exp. Cell Res.*, (in press).

LEITH, J. T., SCHILLING, W. A. & WHEELER, K. T. (1975) Cellular radiosensitivity of a rat brain tumor. *Cancer*, **35**, 1545.

LORD, E. M. (1980) Comparison of *in situ* and peripheral host immunity to syngeneic tumours employing the multicellular spheroid model. *Br. J. Cancer*, **41** (Suppl. IV), 123.

LORD, E. M. & KENG, P. C. (1980) Effects of radiation on *in situ* host cells separated from a murine tumor by centrifugal elutriation. *Radiat. Res.*, **83**, 456.

MEISTRICH, M. L., MEYN, R. E. & BARLOGIE, B. (1977) Synchronization of mouse L-P59 cells by centrifugal elutriation separation. *Exp. Cell Res.*, **105**, 169.

ROCKWELL, S. (1977) *In vivo–in vitro* tumor systems: New models for studying the response of tumors to therapy. *Lab. Anim. Sci.*, **27**, 831.

ROCKWELL, S. (1980) *In vivo–in vitro* tumour cell lines: Characteristics and limitations as models for human cancer. *Br. J. Cancer*, **41** (Suppl. IV), 118.

ROCKWELL, S. C. & HAHN, G. M. (1974) An assay permitting quantitative comparison of tumor-directed immunity and tumor cell survival. *J. Natl Cancer Inst.*, **53**, 1379.

ROCKWELL, S. C., KALLMAN, R. F. & FAJARDA, L. F. (1972) Characteristics of a serially transplanted mouse mammary tumor and its tissue-culture-adapted derivative. *J. Natl Cancer Inst.*, **49**, 735.

ROSENBLUM, M. L., KNEBEL, K. D., WHEELER, K. T., BARKER, M. & WILSON, D. B. (1975) Development of an *in vitro* chemotherapy of a rat brain tumor. *In Vitro*, **11**, 264.

RUSSELL, S. W., DOE, W. F., HOSKINS, R. G. & COCHRANE, D. G. (1976) Inflammatory cells in solid murine neoplasms. I. Tumor disaggregation and identification of constituent inflammatory cells. *Int. J. Cancer*, **18**, 322.

SCHMIDEK, H. H., NIELSEN, S. L., SCHILLER, A. L. & MESSER, J. (1971) Morphological studies of rat brain tumors induced by N-nitrosomethylurea. *J. Neurosurg.*, **34**, 335.

SIEMANN, D. W. & SUTHERLAND, R. M. (1980) *In vivo* tumour response to single and multiple exposures of adriamycin. *Eur. J. Cancer*, **16**, 1433.

STEPHENS, T. C., CURRIE, G. A. & PEACOCK, J. H. (1978) Repopulation of $\gamma$-irradiated Lewis lung carcinoma by malignant cells and host macrophage progenitors. *Br. J. Cancer*, **38**, 573.

STEPHENS, T. C. & PEACOCK, J. H. (1977) Tumour volume response, initial cell kill and cellular repopulation in B16 melanoma treated with cyclophosphamide and 1-(2-chlorotehyl)-3-cyclohexyl-1-nitrosourea. *Br. J. Cancer*, **36**, 313.

STEWART, C. C. & BEETHAM, P. L. (1978) Cytocidal activity and proliferation ability of macrophages infiltrating the EMT6 tumor. *Int. J. Cancer*, **22**, 152.

THOMSON, J. E. & RAUTH, A. M. (1974) An *in vitro* assay to measure the viability of KHT tumor cells not previously exposed to culture conditions. *Radiat. Res.*, **58**, 262.

TWENTYMAN, P. R. (1980) Experimental chemotherapy studies: Intercomparison of assays. *Br. J. Cancer*, **41** (Suppl. IV), 279.

TWENTYMAN, P. R. & YUHAS, J. M. (1980) Use of a bacterial neutral protease for disaggregation of mouse tumours and multicellular tumour spheroids. *Cancer Letters*, **9**, 225.

WHEELER, K. T. (1981) Factors influencing the response of experimental brain tumours to therapy. *Cancer Treat. Reps.*, (in press).

WHEELER, K. T., TEL, N., WILLIAMS, M. E., SHEPPARD, S., LEVIN, V. A. & KABRA, P. (1975) Factors influencing the survival of rat brain tumor cells after *in vitro* treatment with 1,3-bis (2-chloroethyl)-1-nitrosourea. *Cancer Res.*, **35**, 1464.

WHEELER, K. T. & WALLEN, C. A. (1980) Is cell survival a determinant of the *in situ* response of 9L tumours. *Br. J. Cancer*, **41** (Suppl. IV), 299.